# Discipline-specific responses to a complex migraine case: a vignette-based survey among neurologists, psychiatrists, and family physicians

**DOI:** 10.3389/fneur.2025.1646114

**Published:** 2025-09-15

**Authors:** Rahşan Karacı, Esra Aydın Sünbül, Nil Tekin, Bahar Taşdelen, Ikbal Humay Arman, Füsun Mayda Domaç, Aynur Özge

**Affiliations:** ^1^Department of Neurology, Erenköy Psychiatry and Neurological Diseases Training and Research Hospital, University of Health Sciences, Istanbul, Türkiye; ^2^Departments of Psychiatry Erenköy Psychiatry and Neurological Diseases Training and Research Hospital, University of Health Sciences, Istanbul, Türkiye; ^3^Department of Family Medicine, Izmir Faculty of Medicine, Tepecik Research and Training Hospital, Health Sciences University, Izmir, Türkiye; ^4^Department of Biostatistics and Medical Informatics, Medical Faculty, Mersin University, Mersin, Türkiye; ^5^Department of Family Medicine, Faculty of Medicine, Istanbul Medipol University, Istanbul, Türkiye; ^6^Departments of Neurology, Erenköy Psychiatry and Neurological Diseases Training and Research Hospital, University of Health Sciences, Istanbul, Türkiye; ^7^Department of Neurology, Faculty of Medicine, NOROM Neuroscience and Excellence Center, Mersin University, Ankara, Türkiye

**Keywords:** chronic migraine, medication overuse headache, multimorbidity, interdisciplinary care, psychiatry, family medicine, headache management

## Abstract

**Background:**

Chronic migraine (CM), especially when complicated by medication overuse headache (MOH), frequently coexists with psychiatric and somatic comorbidities that challenge conventional monodisciplinary management. Integrated, interdisciplinary care has been proposed as a solution, but real-world implementation remains limited.

**Objective:**

To evaluate and compare the diagnostic reasoning, treatment preferences, and follow-up strategies among neurologists, psychiatrists, and family physicians when managing a complex case of CM with comorbidities and medication overuse.

**Methods:**

A case-based, multidisciplinary study was conducted using a structured vignette of a middle-aged woman with CM + MOH and multiple comorbidities. Ten questions were asked for each specialty (neurologists, psychiatrists, and family physicians) across Türkiye. Responses from 305 clinicians were analyzed via inductive thematic analysis and domain-specific agreement metrics.

**Results:**

Neurologists prioritized headache semiology and pharmacological treatment; psychiatrists emphasized psychosocial burdens and behavioral interventions; and family physicians reported heterogeneous decision-making shaped by system-level constraints. Agreement levels varied by discipline and clinical domain. The level of awareness of multimorbidity was high, yet interdisciplinary coordination was limited. Across groups, common barriers included stigma, poor treatment adherence, and unclear referral pathways.

**Conclusion:**

CM + MOH patients with multimorbidity constitute a clinically complex population requiring interdisciplinary collaboration. The differences in approach highlight the need for structured care pathways and shared decision-making frameworks. Family physicians can act as pivotal coordinators if supported by headache-specific training and referral networks.

## Introduction

Chronic migraine (CM), particularly when complicated by medication overuse headache (MOH), is a highly disabling neurological disorder characterized not only by frequent and intense headache attacks but also by substantial comorbidity burdens. Epidemiological evidence consistently shows that CM is rarely an isolated condition. Instead, it frequently coexists with a variety of physical and mental health conditions, such as anxiety, depression, fibromyalgia, irritable bowel syndrome, and allergic disorders, often constituting a complex multimorbid profile that challenges diagnosis, treatment, and follow-up (1, 2). In particular, the presence and clustering of such comorbidities have been associated with a significantly elevated risk of progression from episodic to chronic forms, higher relapse rates following detoxification interventions, and increased socioeconomic burden (3–5).

Multimorbidity, the co-occurrence of two or more chronic health conditions, has gained traction in headache medicine. However, its application remains inconsistent due to definitional and methodological heterogeneity in the literature (6). Recent meta-analytic data suggest that mental health comorbidities, particularly depression and anxiety disorders, are overrepresented in individuals with migraine, far exceeding their expected population prevalence (2). This calls for a broader clinical lens when evaluating and managing patients with CM, especially in the presence of overlapping behavioral, psychological, and somatic complaints.

Recognizing this complexity, interdisciplinary care models have been increasingly emphasized. In a previous publication by our group, we proposed and demonstrated the utility of a collaborative consultation framework in the management of neuropsychiatric cases that do not fit neatly within single-specialty paradigms. This work, titled “*One Patient, Three Providers*,” involved neurologists, psychiatrists, and family physicians engaging in real-time dialog around complex presentations, allowing shared insights to foster more accurate diagnoses and personalized management plans (7). The collaborative experience highlighted the value of transcending disciplinary silos, particularly in cases where symptoms span the neurological, psychiatric, and primary care domains.

This study aims to describe the clinical heterogeneity of CM + MOH and illustrate how coordinated interprofessional communication can bridge care gaps and improve treatment outcomes in real-life headache practice. Building on this experience, the current study presents a focused, structured, multidisciplinary investigation into a middle-aged case of chronic migraineurs experiencing somatic and psychological symptoms, medication overuse, and resistance to traditional preventive strategies. By gathering the perspectives of specialists and senior trainees in neurology, psychiatry, and family medicine through different discipline-specific question sets, we aim to explore the nuances of diagnostic reasoning, treatment planning, and challenges in caring for complex patients, as well as the importance of a multidisciplinary approach.

## Method

### Study design and setting

This study was a multidisciplinary, qualitative investigation using a structured case vignette and a series of discipline-specific question sets. The aim was to gather and compare expert opinions from neurology, psychiatry, and family medicine on a clinically complex patient with chronic migraine and medication overuse, along with multiple somatic and psychological comorbidities. The study was conducted between April 2025 and May 2025 under the coordination of a tertiary neurology department experienced in interdisciplinary headache care. We collected answers from these three specialties by administering an online case history-based survey that included 10 questions. We aimed to examine how clinicians from different specialties interpreted and managed a complex case of chronic migraine with comorbid medication overuse (CM+MOH). The study hypothesis was generated by asking different specialty-specific questions for the same case, given that clinician responses varied in terms of training, clinical roles, and exposure to headache disorders. These differences were expected to reveal discipline-specific strengths and training needs.

### Case vignette

A representative case was constructed based on real-world headache clinical experience and refined through expert consensus.

### Case

A 49-year-old female sales manager, married with two children, presented to the outpatient clinic with progressively worsening headache attacks that began during high school. In recent months, she reported 15–20 headache days per month, typically starting unilaterally on the left and becoming diffuse, pulsating, or dull, lasting 10–12 h, and partially relieved by sleep. She rated the average pain intensity as 8/10. Attacks are often accompanied by nausea, vomiting, photophobia, and occasionally unilateral lacrimation. The headaches worsened with movement and were not preceded by aura. She also reported scalp sensitivity during attacks, describing discomfort when washing her face or tying her hair, leading her to cut her hair short. Disability scores were high: HIT-6 = 73, MIDAS = 123, and MIBS-4 = 12. The medical history included allergic rhinitis, irritable bowel syndrome, and a 20-pack-year smoking history. The patient’s family history revealed similar complaints from her mother and brother; her mother’s headaches improved during the postmenopausal period, which the patient hoped would have occurred in her case as well. The results of the neurological examination and laboratory workup were unremarkable. Previous treatments included propranolol, flunarizine, amitriptyline, and topiramate, all of which were discontinued by the patient due to side effects. The patient had frequent emergency room visits and was taking 15 nonsteroidal anti-inflammatory drug (NSAID) tablets and 6–8 triptan tablets per month without current preventive medication. Additional issues included problematic caffeine use, anxiety symptoms, and sleep latency disturbances. She refused a referral for psychiatric evaluation.

Importantly, the diagnosis of chronic migraine and medication overuse headache was predefined according to ICHD-3 criteria and embedded in the case vignette. All participants were instructed to respond as if a neurologist or headache specialist had previously diagnosed the patient. Psychiatrists and family physicians were therefore not expected to make a new diagnosis, but rather to reflect on how they would contribute to the management of such a referred patient.

### Questionnaire development

Senior academic experts in neurology, psychiatry, and family medicine independently developed a set of 10 structured questions for each of the three specialties. The questions were designed to assess each discipline’s preferences in diagnosing, treating, and following up on a complex chronic migraine case with comorbidities. Additional items addressed comorbidity assessment and perceived system-level challenges. The content validity of each question set was reviewed through iterative feedback among the research team. The final versions of the questionnaires are included in Supplementary Files 1–3.

To enhance ecological validity and align with the clinical responsibilities of each specialty, the surveys were tailored accordingly: neurologists received a fully vignette-based questionnaire; psychiatrists received one vignette-based question (Q7) in addition to general questions related to management, comorbidities, and collaboration; and family physicians received a version focused on broader decision-making patterns and perceived challenges in headache care, without specific case-based items. While this design allowed us to capture discipline-specific insights, we acknowledge that it may limit direct comparability between groups. We want to point out that this methodological limitation is further addressed in the discussion.

### Participant selection and data collection

Experts and senior residents (PGY-4 and above) from academic institutions across Türkiye were invited to participate. Although we did not collect data on clinicians’ participation in headache-specific education (e.g., courses or congresses), we included their years of professional experience and institutional setting to provide context for their responses. This approach reflects our understanding that structured headache training is not uniformly expected across all disciplines, particularly among psychiatrists and family physicians. All participants were informed of the study’s purpose, and informed consent was obtained electronically. We acknowledge that gender and professional background can shape perceptions and clinical choices, as highlighted in similar workforce studies (8).

### Data analysis

An inductive thematic analysis approach was used to identify recurring patterns and discipline-specific priorities. Two independent researchers performed initial coding, which was then compared and reconciled through discussion with a third reviewer. Codes were clustered into overarching themes such as “diagnostic convergence,” “treatment divergences,” “psychological burden,” and “barriers to interdisciplinary coordination.” Quantitative summaries (e.g., rankings of treatment priorities) were tabulated where applicable. The percentages of correct answers in each group were compared via a Z-test for two proportions. Microsoft Excel and JASP were used for data analysis and graphical illustrations.

### Ethical considerations

The study protocol was approved by the Clinical Research Ethics Committee of Medipol University, İstanbul (Approval Number: 435), and adhered to the principles outlined in the Declaration of Helsinki. All the data were anonymized, and the participants provided informed consent to use their responses in research dissemination.

## Results

### Participants and demographics

A total of 305 participants contributed to the study: 101 neurologists, 100 psychiatrists, and 104 family physicians. The sample included senior residents and attending faculty from academic hospitals and primary care centers across Türkiye.

Table 1 presents the demographic and professional characteristics of the clinicians participating in the survey. All participants responded to a standardized vignette involving a pre-diagnosed case of chronic migraine with medication overuse and multimorbidity, rather than making an independent diagnosis. Years of experience and institutional settings were collected to reflect the level of professional exposure, particularly since headache-specific training is not uniformly expected across all disciplines.

**Table 1:** Demographic characteristics of the participants by specialty: thematic analysis across disciplines.

Variable	Neurology (*n* = 101)	Psychiatry (*n* = 100)	Family medicine (*n* = 104)
Age (years)	41.6 ± 7.5	35.3 ± 6.5	37.4 ± 10.5
Years of experience	10.7 ± 7.2	6.0 ± 5.1	8.7 ± 8.4
Female	75 (74.3%)	62 (62.0%)	60 (57.7%)
Male	26 (25.7%)	38 (38.0%)	44 (42.3%)

### Diagnostic reasoning and domain-specific trends

#### Neurologists

**Figure 1:**
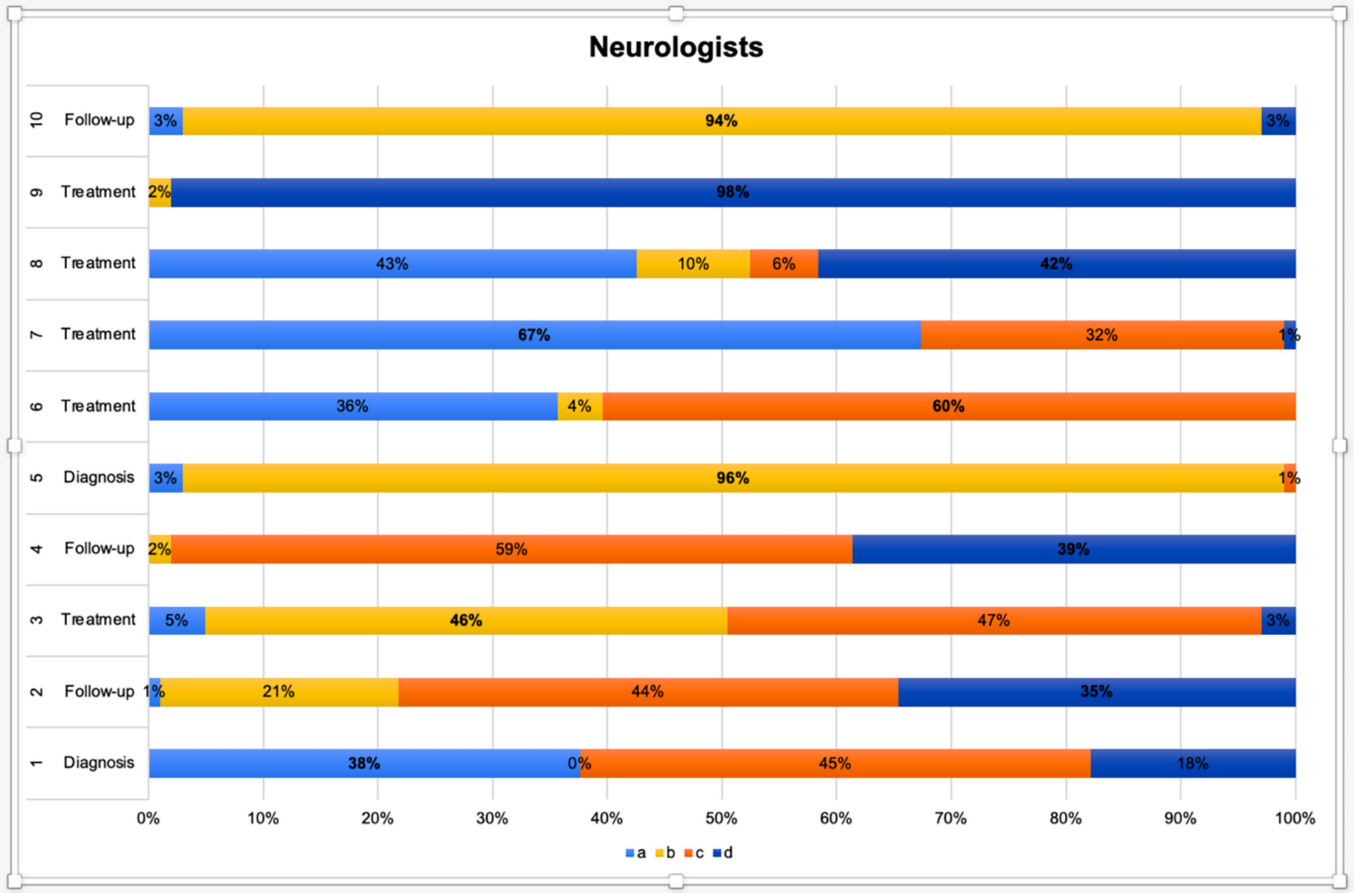
Distribution of neurologists’ responses across diagnostic, follow-up, and treatment domains.

The following subsection presents findings specifically from neurologists, acknowledging that results are interpreted within the context of this discipline and not meant for direct comparison. Neurologists emphasized classical migraine features such as unilateral pain, pulsatile quality, and associated symptoms like photophobia and nausea. They showed the highest alignment with guideline-based diagnostic markers, and their answers frequently referred to ICHD-3 criteria. In the follow-up and treatment domains, they prioritized pharmacological strategies and structured preventive approaches. Agreement among neurologists was particularly high in questions related to diagnosis and planning of prophylactic treatment (Q5, Q9, Q10). Figure 1 displays their response distribution.

This figure presents the distribution of neurologists’ responses to 10 clinical decision-making scenarios categorized under the diagnosis, follow-up, and treatment domains. Each horizontal bar represents one item (from Q1 to Q10), with stacked segments indicating the percentage of respondents selecting options *a*, *b*, *c*, or *d*. Color codes correspond to the answer choices:

- *a* (light blue),
- *b* (yellow),
- *c* (orange),
- *d* (dark blue).

#### Psychiatrists

**Figure 2:**
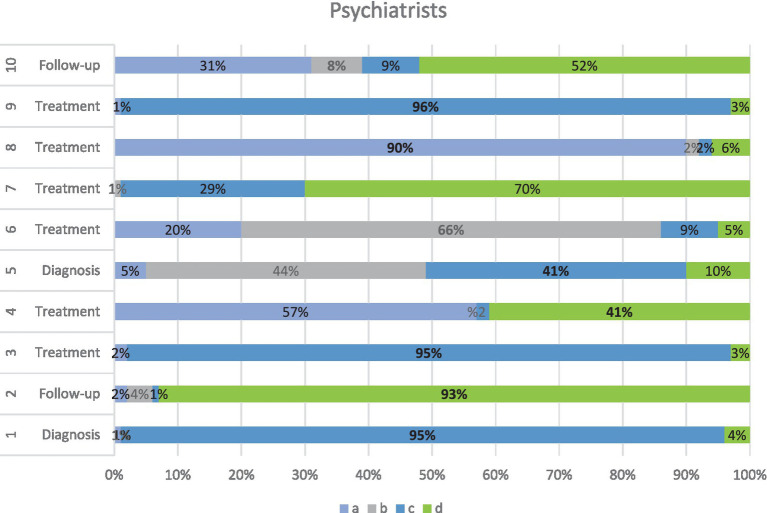
Distribution of psychiatrists’ responses across diagnostic, follow-up, and treatment domains.

Psychiatrists’ responses are reported here as a separate group to respect the tailored structure of their survey tool. Psychiatrists focused more on the psychological burden and affective comorbidities. They gave significant weight to disability scores (HIT-6, MIDAS), mood symptoms, and patient coping behaviors. Behavioral therapy and antidepressant use were frequently selected in treatment strategies. While diagnostic agreement was moderate, a high level of consensus was observed regarding the importance of psychosocial follow-up. Figure 2 illustrates their domain-specific responses.

This horizontal stacked bar chart illustrates how psychiatrists responded to 10 clinical vignettes categorized under the diagnosis, follow-up, and treatment domains in the management of chronic migraine. Each bar represents the percentage of participants selecting options *a* (light blue), *b* (light gray), *c* (dark blue), or *d* (green). The diversity of responses reflects differing clinical approaches across items, with high consensus observed for items Q1, Q2, Q3, Q8 and Q9 (≥ 90% agreement). Notably, reactions in the follow-up and treatment domains were more variable, indicating a broader range of psychiatric perspectives on multidisciplinary migraine care.

#### Family physicians

**Figure 3:**
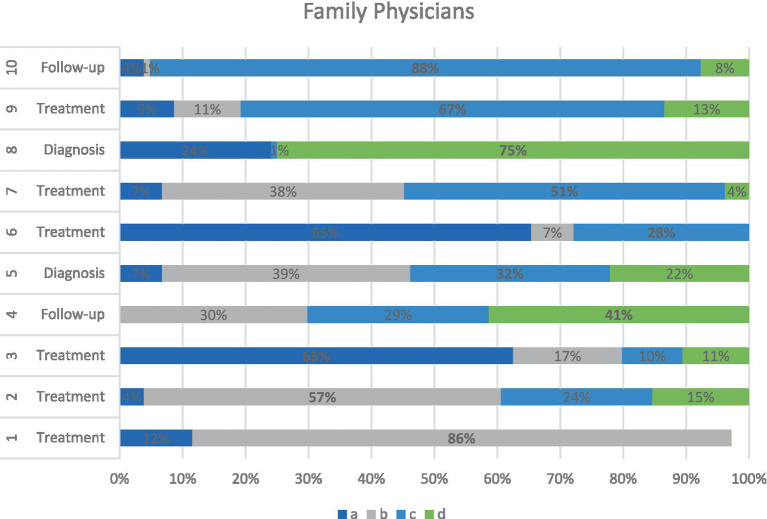
Distribution of family physicians’ responses across diagnostic, follow-up, and treatment domains.

Family physicians’ section provides insight into generalist perspectives, analyzed independently due to differences in survey format. Family physicians demonstrated the most variable patterns in diagnosis and treatment. They often based decisions on symptom severity and systemic limitations (e.g., time constraints, access to specialists). Their responses reflected a pragmatic approach: some focused on patient education and lifestyle interventions, while others emphasized the need for referral. Notably, they scored lower on diagnostic accuracy based on predefined key responses but showed higher consistency in follow-up-related decisions. Figure 3 outlines these findings.

This horizontal stacked bar chart illustrates the response patterns of family physicians to 10 clinical items, classified into the diagnosis, follow-up, and treatment domains. Each bar reflects the percentage of participants selecting each of the four multiple-choice options: *a* (dark blue), *b* (gray), *c* (light blue), and *d* (green). While some questions (e.g., Q1, Q10) show dominant agreement (≥ 85%) on a single option, other questions reveal a more varied distribution, indicating greater uncertainty or divergent approaches within the group. Compared with other specialties, family physicians demonstrated the most heterogeneous patterns in treatment-related decision-making, reflecting the broad scope and referral-based nature of their clinical roles.

##### Treatment attitudes

Consensus varied significantly across groups. Neurologists favored evidence-based chemoprophylaxis, whereas psychiatrists leaned toward integrative models involving behavioral therapy and antidepressants. Family physicians exhibited the most heterogeneous treatment selections, which were often influenced by systemic constraints and patient adherence concerns (Figure 3).

#### Quantitative consensus patterns

**Figure 4:**
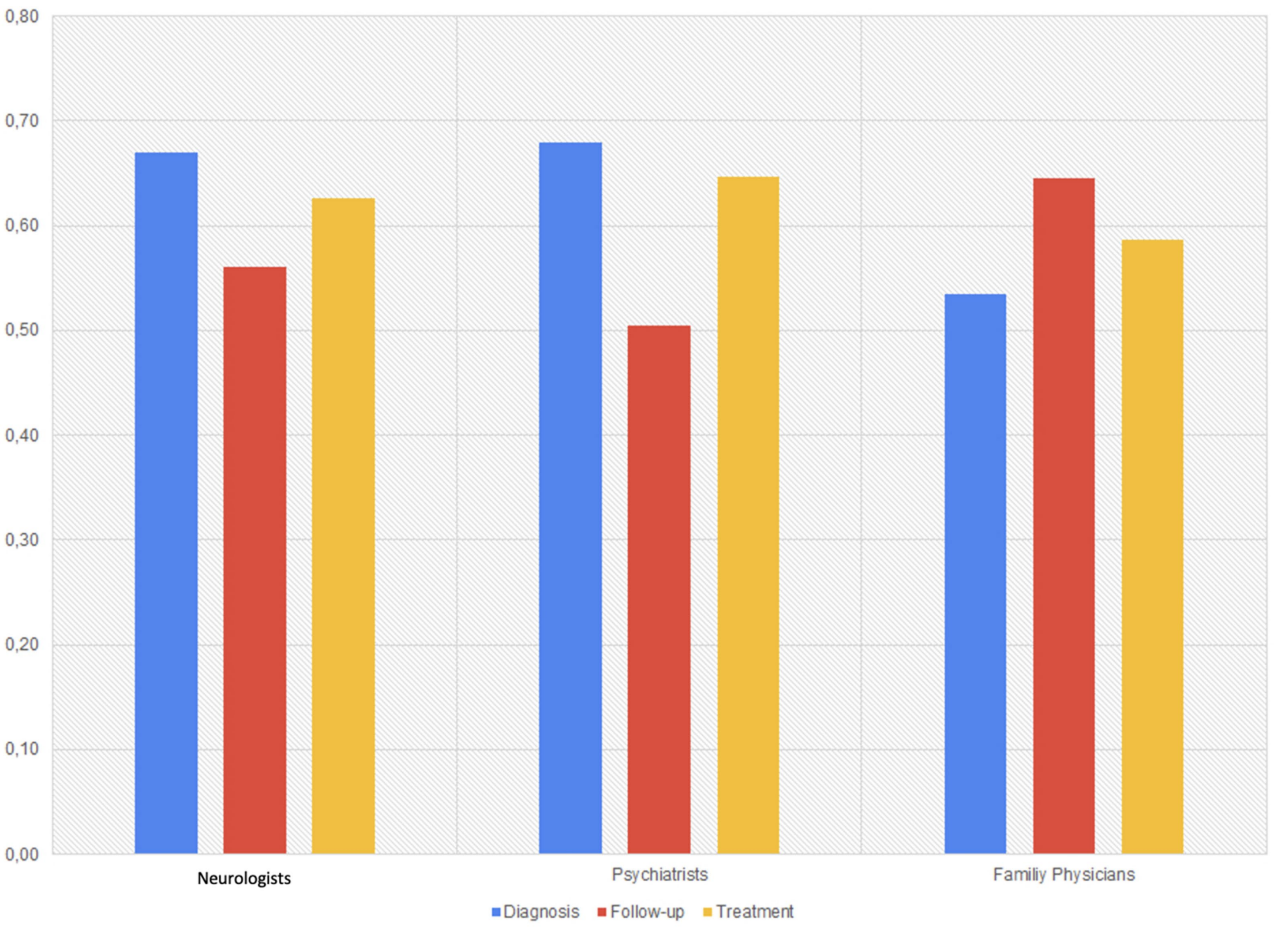
Mean domain-specific agreement scores by specialty group.

Across all groups, domain-specific agreement scores revealed interesting divergences (Figure 4). Spearman correlation analyses were performed to explore whether participant age or years of professional experience influenced response accuracy. However, no significant correlation was found between either variable or the percentage of correct answers in any domain (*p* > 0.05), with correlation coefficients near zero.

To highlight patterns within each discipline rather than imply direct comparisons, we examined domain-specific agreement levels (Figure 4). This figure presents within-discipline response patterns across selected domains. Due to the tailored nature of the survey, no direct statistical comparison was performed across groups.

- Psychiatrists showed the highest alignment in diagnostic reasoning (mean agreement score: 0.68), often guided by psychological frameworks.
- Family physicians showed the highest agreement in follow-up strategies, possibly reflecting consistency in managing chronic patients in primary care.
- Neurologists demonstrated strongest agreement in treatment planning, with a pharmacological and structured approach.

#### Multidisciplinary overlaps and divergences

Despite different priorities, all groups acknowledged the challenges of managing CM + MOH with multimorbidity’s. Common barriers included:

- Stigma, especially regarding psychiatric comorbidities;
- Poor adherence to preventive strategies;
- Unclear or inconsistent referral mechanisms.

Family physicians often reported structural barriers such as insufficient headache training and limited access to neurologists. Psychiatrists described diagnostic ambiguity and fragmented care. Neurologists cited lack of integrated support and delayed referrals.

These findings suggest a shared interest in improving interdisciplinary coordination, though specific challenges and needs vary by specialty.

## Discussion

### Overview and interpretation of the key findings

This study provides meaningful insights into the clinical complexity of patients with CM and MOH, particularly when CM and MOH cooccur with multiple somatic comorbidities. The key findings indicate that multimorbidity is not only highly prevalent in this population but also significantly associated with higher relapse rates and increased treatment burden.

In alignment with previous findings from D’Amico et al., our study reinforces the evidence that patients with CM + MOH frequently present with multiple chronic conditions that compound the overall disease burden and complicate treatment trajectories (9). Notably, a significant proportion of our sample had more than three comorbidities, a threshold often used to define complex multimorbidity (6). This finding highlights the necessity for comprehensive patient assessments and integrated care approaches.

Moreover, our results support and extend the conclusions from the CaMEO study, which demonstrated that the presence and constellation of comorbidities substantially influence the risk of progression from episodic migraine to CM (3). Patients classified in comorbidity-rich clusters presented elevated hazard ratios for chronification, even after adjustment for clinical severity markers such as MIDAS and medication overuse, underscoring the independent impact of the comorbidity burden (3).

Consistent with previous longitudinal studies, relapse after withdrawal treatment remains a critical issue. In our cohort, nearly one in three patients relapsed within the follow-up period, mirroring the relapse rates reported by Raggi et al., who reported that depression and somatic complaints were among the strongest predictors of poor long-term outcomes (4). This overlap is crucial, as it supports the need for early psychosocial and psychiatric assessment in CM + MOH populations.

Additionally, economic implications cannot be overlooked. The literature shows that MOH is associated with substantial direct and indirect costs (5, 10). The high frequency of health care utilization and the need for multidisciplinary interventions in patients with multimorbidity intensify this economic burden.

Finally, qualitative findings by Scaratti et al. and Raggi et al. have shown that frequent relapses not only carry heavier symptom loads but also face higher levels of psychosocial distress, stigmatization, and workplace dysfunction (4.11). Our clinical observations confirm these psychosocial complexities, especially in patients with overlapping respiratory, cardiovascular, and psychiatric conditions—again underscoring the urgent need for individualized, biopsychosocial care models (11).

### Value of the multidisciplinary approach

The findings of this study underscore the significant added value of adopting a multidisciplinary approach in the management of chronic migraine with medication overuse and comorbid conditions. The complexity of these cases—often involving overlapping psychiatric, gastrointestinal, respiratory, and lifestyle-related factors—requires nuanced interpretation and personalized management strategies that are rarely achievable within the confines of a single discipline.

Importantly, our results support the working hypothesis that clinicians from different disciplines will exhibit different patterns of clinical response due to their varying roles, training, and exposure. Neurologists excelled in diagnostic precision and pharmacological planning, while psychiatrists emphasized emotional comorbidities and behavioral contributors. Family physicians demonstrated a broader, more holistic perspective, often more readily incorporating systems-based and lifestyle interventions. Differences in the responses of neurologists, psychiatrists, and family physicians are presented not as statistical comparisons across disciplines, but rather as discipline-specific response patterns. This allowed us to highlight complementary strengths and gaps across specialties.

These findings are consistent with previous qualitative research showing that chronic migraine patients with frequent relapses tend to benefit more from integrative care models that consider not only the biomedical aspects of pain but also the psychosocial context and healthcare navigation challenges (11, 12). Such convergence helps reduce care fragmentation, ensures a more comprehensive evaluation of comorbidities, and facilitates shared decision-making that aligns with patient expectations and preferences.

Moreover, longitudinal studies suggest that addressing multimorbidity and medication overuse within a unified care plan may improve long-term outcomes, including reduced relapse rates and healthcare costs (5, 10). Therefore, establishing interdisciplinary headache teams or structured referral pathways between neurology, psychiatry, and primary care may provide a sustainable model for managing high-risk migraine patients.

In summary, the multidisciplinary model not only facilitates more accurate assessment and tailored treatment but also aligns with current understanding of migraine as a multisystem disorder with fluctuating biological and psychosocial dimensions. Such collaboration is beneficial and necessary for patients who have failed monodisciplinary care or who carry a high comorbidity burden.

### Clinical implications

The results of this study have important clinical implications for the management of chronic migraine, particularly in patients with medication overuse and multimorbidity. The heterogeneity observed across disciplines in treatment preferences, follow-up practices, and prioritization of comorbidities underscores the necessity of shifting from fragmented, specialty-specific care to coordinated, patient-centered strategies.

Our data revealed discipline-specific barriers and opportunities: neurologists cited diagnostic clarity and under-referral; psychiatrists emphasized late-stage involvement and lack of structured communication; general practitioners reported insufficient training and limited access to specialist care. These patterns suggest targeted areas for educational investment and the development of healthcare policy.

First, clinicians need to recognize that chronic migraine is rarely an isolated neurological disorder. Large-scale studies have shown that comorbidities, including psychiatric, gastrointestinal, or allergic conditions, significantly modify the disease course and treatment response (1). Integrating psychiatric screening tools, dietary reviews, and sleep assessments into neurological consultations may help identify contributing factors that traditional headache evaluations may miss.

Second, early identification and management of medication overuse—a common feature in our case and respondent profiles—are crucial. Evidence shows that simple withdrawal alone may be insufficient without concurrent behavioral, psychiatric, and social support interventions (10, 11). Embedding behavioral specialists and mental health support in migraine clinics or ensuring close collaboration with psychiatric and family medicine may reduce relapse rates and optimize adherence to preventive strategies.

Third, our findings reflect distinct educational needs across clinician groups. Neurologists requested more tools to manage psychiatric comorbidities; psychiatrists indicated a need for structured headache education; family physicians asked for practical, referral-focused updates. Tailoring training to each group while promoting shared learning opportunities may enhance interdisciplinary efficiency.

Moreover, the divergence in response patterns across disciplines highlights the need for shared educational initiatives and clinical decision aids to bridge the gaps in training and treatment familiarity. For example, developing consensus-based flowcharts for diagnosing and managing complex migraine presentations may help non-neurology specialists deliver more confident and guideline-concordant care.

Family physicians are well-positioned to play a central role in longitudinal monitoring, patient education, and specialist coordination. However, our findings suggest that these patients may benefit from targeted updates in migraine pharmacotherapy, including triptan use, CGRP-based treatments, and nonpharmacological adjuncts. Strengthening their role through structured headache education and referral networks could significantly increase healthcare system efficiency and patient satisfaction.

Finally, these insights support the establishment of interdisciplinary care pathways, such as shared electronic health records, multidisciplinary case conferences, or collocated services, that facilitate timely and cohesive care across neurology, psychiatry, and primary care. Such systemic changes are likely to yield both clinical and economic benefits, as previously demonstrated in real-world analyses of MOH and chronic migraine care (5, 10).

### Strengths

One of this study’s key strengths lies in its innovative, real-world simulation approach. This approach uses a detailed, clinically representative case to explore divergent and convergent responses from three distinct medical disciplines: neurology, psychiatry, and family medicine. This design allows for a more ecologically valid understanding of how complex chronic migraine with medication overuse and comorbidities is perceived and managed in practice. The structured, question-based evaluation ensured standardized input from each group while allowing for discipline-specific variability to emerge.

Furthermore, the study builds upon and complements prior multidisciplinary frameworks proposed in earlier collaborative work (7), offering more profound insight into specialty-specific decision-making in migraine care. The inclusion of faculty-level experts and experienced senior residents increases the credibility of the responses and reflects the diversity of clinical perspectives within each field.

### Limitations

Despite its strengths, the study has several limitations. First, the case-based nature of the survey, while rich in clinical detail, may not capture the full range of diagnostic and management variability encountered in everyday clinical settings. Responses were based on a single vignette, and generalizability to other migraine subtypes or clinical scenarios (e.g., menstrual migraine, posttraumatic headache) may be limited.

Second, although the participant pool was highly populated by clinicians, we did not include specific items regarding participation in headache-focused training or continuing education. We acknowledge that this limits our ability to interpret the influence of clinician specialty on response patterns and emphasize that this should be considered in future survey-based studies. The sample size within each specialty was modest and geographically limited, limiting broader generalizability. The absence of allied health professionals (e.g., clinical psychologists, dietitians, headache nurses) in this interdisciplinary assessment is another notable gap, as these professionals play increasingly recognized roles in multimodal headache care.

Third, although the survey explored diagnostic, follow-up, and treatment decisions, it did not assess the reasons behind individual responses or capture contextual factors (e.g., healthcare system constraints, medication access, patient preferences) that may influence decision-making. The inclusion of qualitative interviews or focus groups in future studies could enhance interpretability and inform more tailored implementation strategies.

Importantly, while providing differently structured questionnaires to all participant groups may have increased ecological validity, it may have potentially compromised cross-specialty comparability in framing clinical challenges.

Finally, as with all self-reported assessments, the possibility of social desirability bias or theoretical rather than practical responses cannot be excluded. However, using an authentic, complex patient scenario helps mitigate this risk by anchoring responses in a clinically realistic framework.

### Future directions

This study highlights several promising avenues for future research and clinical innovation in the care of patients with chronic migraine and medication overuse, especially those with multimorbid presentations.

First, future studies should explore the implementation of structured multidisciplinary care models in real-world settings. Longitudinal evaluations of multidisciplinary headache clinics—incorporating neurology, psychiatry, family medicine, and behavioral health—could assess their impact on outcomes such as relapse rates, quality of life, healthcare utilization, and treatment adherence. Comparative studies between traditional mono-specialty and collaborative care pathways would offer valuable insight into cost-effectiveness and scalability (5, 10).

Second, expanding the scope of participant specialties to include headache nurses, clinical psychologists, pharmacists, and physiotherapists could provide a more holistic understanding of team-based care and delineate each professional’s role in optimizing patient outcomes. Their inclusion in future surveys or consensus panels would also enhance interdisciplinary alignment and care coordination.

Third, qualitative research, such as focus groups or semistructured interviews with clinicians and patients, can illuminate the decision-making dynamics, barriers to referral or collaboration, and unmet educational needs identified in this study. Insights from such work could inform the design of interdisciplinary guidelines or care algorithms tailored for patients with complex migraine presentations.

Fourth, the development of validated interdisciplinary training modules should be prioritized. Based on the apparent divergence in our findings, educational interventions must be both discipline-specific and collaborative, targeting neurologists’ need for psychosocial integration, psychiatrists’ need for headache knowledge, and GPs’ need for practical diagnostic and referral tools. Interactive, case-based formats may facilitate mutual understanding and build confidence across roles.

Additionally, digital health tools, such as integrated decision support systems or telecollaboration platforms, could be piloted to bridge the gap between specialties, particularly in resource-limited settings. These tools may help generalists and psychiatrists apply evidence-based migraine care while maintaining timely referral pathways to specialized centers.

Finally, the development and validation of shared clinical decision-making tools or multidisciplinary training modules—based on the diagnostic, follow-up, and treatment discrepancies revealed in this study—could empower a wider group of providers to manage migraine patients more confidently and effectively.

In summary, this study opens the door to broader rethinking of migraine care delivery, calls for expanded collaboration, innovative care models, and further research into the real-world impact of multidisciplinary integration.

## Conclusion

This study highlights the importance of a multidisciplinary approach in managing chronic migraine complicated by medication overuse and comorbid conditions. Through a structured case-based assessment involving neurologists, psychiatrists, and family physicians, we identified overlapping and divergent clinical perspectives reflecting each specialty’s strengths. These findings underscore the need for integrated, patient-centered care models to address the complexity of migraine management and support the transition from fragmented practices to more coordinated and effective treatment pathways.

## Data Availability

The datasets presented in this study can be found in online repositories. The names of the repository/repositories and accession number(s) can be found in the article/Supplementary material.
