# Supplementary material for: Discipline-specific responses to a complex migraine case: a vignette-based survey among neurologists, psychiatrists, and family physicians
Source: Front Neurol. 2025 Sep 15;16:1646114. doi: 10.3389/fneur.2025.1646114 (PMC12476989; doi:10.3389/fneur.2025.1646114)
Supplement: Supplementary file 1 [file Data_Sheet_1.zip › Supplementary Material/Data Sheet_1.DOCX]

Neurology questionaire

1-Which of the following is the most striking feature of the patient's headaches that would allow you to make a diagnosis? (Diagnosis)

a. Unilateral pain

b. Bilateral pain

c. Throbbing

d. Concomitant cranial autonomic findings

2-What is your impression of the patient's Headache Impact Test (HIT-6) and Migraine Disability Assessment (MIDAS) scores? (Follow-up)

a. Both scores indicate mild impairment

b. Both scores indicate moderate impairment

c. HIT-6 indicates severe impairment, while MIDAS indicates moderate impairment

d. HIT-6 indicates moderate deterioration, MIDAS indicates severe deterioration

3-Which of the preventive medication options has caused the patient to gain weight? (treatment)

a. Propranolol

b. Flunarizine

c. Amitriptyline

d. Topiramate

4-What would you expect the patient to do after menopause? (follow-up)

a. Get worse

b. Stay the same

c. Get better

d. All of them

5-What is your suggested diagnosis for the patient's condition? (diagnosis)

a. Chronic migraine

b. Chronic migraine plus medication overuse headache

c. Headache attributed to psychiatric disorders

d. Somatization disorder

6- What lifestyle change would you emphasize as part of the treatment plan? (treatment)

a. Reducing caffeine intake

b. Avoiding alcohol consumption

c. Improving sleep hygiene

d. Increasing salt intake

7-What is the primary reason for the patient's reluctance to accept psychiatric referral? (treatment)

a. Fear of stigma

b. Financial constraints

c. Lack of faith in psychiatric treatment

d. Time constraints due to work

8-What intervention can be recommended to increase the effectiveness of medication during acute attacks? (treatment)

a. Peripheral nerve blocks

b. Cognitive-behavioral therapy

c. Botulinum toxin injections

d. Domperidone administration

9- What would you recommend for the patient's treatment plan? (treatment)

a. Adding an opiate to the attack treatment

b. Using triptans and combined analgesics alternately during attacks

c. Referring to an alternative solution such as cupping therapy, migraine surgery

d. Planning an appropriate teamwork in terms of holistic treatment and starting bridge treatment and prophylaxis

10- What would be the first result you would expect from the treatment? (follow-up)

a. Increased frequency of headaches

b. Decreased frequency and severity of headaches

c. Complete relief of pain

d. Complete relief of sleep disorders and mood problems
