# Supplementary material for: Discipline-specific responses to a complex migraine case: a vignette-based survey among neurologists, psychiatrists, and family physicians
Source: Front Neurol. 2025 Sep 15;16:1646114. doi: 10.3389/fneur.2025.1646114 (PMC12476989; doi:10.3389/fneur.2025.1646114)
Supplement: Supplementary file 1 [file Data_Sheet_1.zip › Supplementary Material/Data Sheet_2.DOCX]

Psychiatry questionaire

1-Which of the following psychiatric conditions is commonly associated with chronic migraine? (Diagnosis)

a. Bipolar disorder

b. Schizophrenia

c. Anxiety disorder

d. Obsessive compulsive disorder

2-Which of the following scales is commonly used to assess the impact of migraine on the daily functioning and emotional health of the patient? (follow-up)

a. Hamilton Depression Rating Scale (HAM-D)

b. Generalized Anxiety Disorder 7-item scale (GAD-7)

c. Beck Depression Inventory (BDI)

d. Migraine Disability Assessment (MIDAS)

3-What is the primary goal of behavioral therapy in the treatment of chronic migraine? (treatment)

a. Relief of symptoms

b. Increasing medication compliance

c. Addressing psychological triggers and coping mechanisms

d. Preventing medication overuse

4-Which pharmacologic approach is frequently used to treat comorbid depression in patients with chronic migraine? (treatment)

a. Selective serotonin reuptake inhibitors (SSRIs)

b. Benzodiazepines

c. Atypical antipsychotics

d. Tricyclic antidepressants

5-What percentage of patients with chronic migraine are estimated to have comorbid depression or anxiety disorders? (Diagnosis)

a. 10-20%

b. 30-40%

c. 50-60%

d. 70-80%

6-Which coping strategy is frequently recommended for patients with chronic migraine to manage stress and anxiety? (treatment)

a. Avoidance

b. Problem solving

c. Rumination

d. Denial

7-What is the primary reason for the patient's reluctance to accept a psychiatric referral in the case study? (treatment)

a. Fear of stigma

b. Financial constraints

c. Lack of faith in psychiatric treatment

d. Time constraints due to work

8-What type of psychotherapy is recommended as an adjunctive treatment for chronic migraine? (treatment)

a. Cognitive-behavioral therapy (CBT)

b. Psychodynamic therapy

c. Interpersonal therapy (IPT)

d. Dialectical behavior therapy (DBT)

9-What intervention is recommended for treating medication overuse in patients with chronic migraine? (treatment)

a. Increasing the dose of analgesics

b. Abrupt discontinuation of all analgesic medications

c. Gradual reduction of analgesic medications

d. Switching to stronger analgesics

10- What is the primary outcome measure used to assess response to treatment in patients with chronic migraine and comorbid depression? (follow-up)

a. Decreased headache frequency

b. Improvement in mood symptoms

c. Decreased drug overuse

d. Increased quality of life
