# Supplementary material for: Discipline-specific responses to a complex migraine case: a vignette-based survey among neurologists, psychiatrists, and family physicians
Source: Front Neurol. 2025 Sep 15;16:1646114. doi: 10.3389/fneur.2025.1646114 (PMC12476989; doi:10.3389/fneur.2025.1646114)
Supplement: Supplementary file 1 [file Data_Sheet_1.zip › Supplementary Material/Data Sheet_3.DOCX]

Family physicians questionaire

1-What is the first-line treatment for acute migraine attacks in primary care? (treatment)

a. Triptans

b. Non-steroidal anti-inflammatory drugs (NSAIDs)

c. Opioids

d. Ergotamines

2-Which lifestyle changes are recommended to prevent migraine attacks in patients with chronic migraine? (treatment)

a. Increased caffeine consumption

b. Regular exercise

c. Quitting smoking

d. Irregular sleep pattern

3-Which drug class is commonly used in the prophylactic treatment of chronic migraine in primary care? (treatment)

a. Beta blockers

b. Antiepileptic drugs

c. Calcium channel blockers

d. Benzodiazepines

4-What is the primary role of family physicians in the treatment of chronic migraine? (follow-up)

a. What is the primary role of family physicians in the treatment of chronic migraine?

b. Prescribing preventive and acute migraine drug therapies

c. Referring patients to specialized headache centers for comprehensive treatment

d. Providing emotional support and education about migraine triggers

5- What percentage of patients with migraine headaches are estimated to have medication overuse headache (MOH)? (Diagnosis)

a. Less than 5%

b. 10-20%

c. 30-40%

d. More than 50%

6-What nonpharmacologic intervention is there evidence to reduce the frequency and severity of migraine attacks? (treatment)

a. Acupuncture

b. Cupping

c. Relaxation

d. Leeching

7-What is the minimum recommended duration of prophylactic treatment for topiramate in chronic migraine? (treatment)

a. 1 month

b. 3 months

c. 6 months

d. 1 year

8- Which comorbid condition is most common in chronic migraine patients and requires close monitoring by family physicians? (diagnosis)

a. Hypertension

b. Diabetes mellitus

c. Hyperthyroidism

d. Depression

9-Which drugs should be avoided in migraine patients due to their potential to aggravate headaches and lead to overuse of medications? (treatment)

a. Acetaminophen

b. Ibuprofen

c. Codeine

d. Naproxen

10- Which should be prioritized in the evaluation of headaches in primary care? (follow-up)

a. Immediate referral to a neurologist

b. Radiologic imaging

c. Headache diary

d. Initiation of prophylactic treatment
